# Harnessing technology in education: The function of RIPPLES model in ICT utilization in public secondary schools of Amhara, Ethiopia

**DOI:** 10.1371/journal.pone.0337796

**Published:** 2025-12-04

**Authors:** Shimelis Gebeyehu Kebede, Melaku Mengistu Gebremeskel, Sifelig Taye Nigatu

**Affiliations:** Department of Educational Planning and Management, Bahir Dar University, Bahir Dar, Ethiopia; University of Johannesburg Faculty of Education, SOUTH AFRICA

## Abstract

This study examined factors influencing the utility of ICT in enhancing teaching and learning effectiveness in secondary schools in the state of Amhara, Ethiopia. It specifically explored the effects of financial resources, infrastructure, personnel, policy, learning, evaluation, and support, as well as teachers’ and students’ perceptions of ICT utility. A correlational research design was employed, involving 739 teachers and 758 students selected through multistage sampling. Data were collected using a questionnaire and then analyzed using confirmatory factor analysis, independent-samples t-tests, and multiple regression analyses. Findings showed that the specified antecedents explained 65.9% and 52.5% of the variance in ICT utility for teachers and students, respectively. Support and access to ICT infrastructure significantly predicted ICT use, while ICT policy was the least influential factor. The t-test indicated a small difference between teachers’ and students’ perceptions (t = 2.771, d = 0.143), with both groups reporting below-average confidence in ICT’s usefulness. The study concludes that while ICT has the potential to enhance teaching and learning in secondary schools, its effective utilization is hindered by inadequate infrastructure, limited support, and weak policy implementation. To maximize ICT’s educational impact, policymakers and school leaders must adopt practical, evidence-based strategies that bridge the gap between policy intentions and classroom realities. They should strengthen technical and administrative support, provide adequate infrastructure, and offer ongoing training. Policymakers and educational leaders must also review ICT policies to ensure alignment with practical implementation needs.

## 1. Introduction

The term information and communication technology (ICT) is commonly used to describe the processes of information processing, modification, and communication. According to [1], it encompasses the tools, applications, networks, and channels that enable the collection, transmission, and processing of information. [2] argues that ICT has become an integral part of modern human life. In particular, it has transformed the education sector by making instructional practices more engaging and efficient [3]. It also provides resources for both traditional and virtual learning environments that foster active and interactive classroom experiences [4,5]. In general, effective ICT integration can significantly enhance students’ academic achievement in schools [6,7].

ICT also promotes both collaboration and communication among students. In this regard, [8] emphasizes that ICT enhances teamwork through project-based learning and international classroom interactions. Similarly, [9] contend that integrating ICT fosters seven key 21st-century skills: technical, informational, communicative, cooperative, critical thinking, creative, and problem-solving. In other words, ICT integration helps students critically evaluate online information, an essential ability in the digital age, although this process requires guided instruction to prevent misinformation [10]. Moreover, [11]argues that employing ICT in schools facilitates assessment and feedback by enabling teachers to tailor instruction to students’ needs.

In the same vein, [6] argue that the growing digital era compels educational systems to integrate ICT as a key strategy to enhance classroom teaching and student learning. According to these authors, ICT integration supports more effective pedagogical approaches, promotes inclusivity, and prepares learners for participation in a digital society. Additionally, ICT enhances student motivation, engagement, and interactive learning while making education more collaborative and visually stimulating [12]. Tools such as interactive whiteboards, educational apps, and multimedia resources make assignments more dynamic; for example, simulations and videos can simplify complex concepts in subjects like science and mathematics [13]. Moreover, [14,15], and [16] emphasize that ICT facilitates personalized or differentiated instruction by allowing students to progress at their own pace, addressing individual learning gaps, and enabling deeper learning through connections between the curriculum and real-world problems.

Recognizing its ability to revolutionize education in developing countries, many have invested substantially in ICT [17]. In recent decades, Ethiopia has launched national initiatives to incorporate ICT into education across primary and higher learning institutions [18–20]. As part of this effort, the Ethiopian government has formulated policies and strategies and dedicated substantial resources to incorporating ICTs into Ethiopia’s education system [21]. However, several studies [4,22,23] indicate that many practical issues persist in the implementation of educational technologies.

### 1.1. Conceptual framework

While many models and theories explain the use of ICT in education, most focus primarily on individual behavior. [24],however, introduced a model that emphasizes institutional elements rather than individual actions. This model, known by the acronym RIPPLES (resources, infrastructure, people, policies, learning, evaluation, and support), is considered one of the most robust, comprehensive, and suitable frameworks for examining the policy and strategic determinants of ICT adoption. It incorporates essential components such as infrastructure, school ICT policy, evaluation methods, and support systems, all of which help assess and promote the effective integration of ICT in education [25]. Unlike other models that primarily highlight individual factors as the main drivers of technology adoption, Benson and Palaskas argue that the RIPPLES model focuses on institutional factors influencing ICT adoption.

According to [26] and [24], resources refer to the budget or financial means available for a project. Sufficient funding is crucial for integrating instructional technology, as acquiring essential ICT infrastructure and learning materials requires financial investment. In many developing countries, incorporating technology into educational systems remains challenging due to the substantial public investment it demands [27]. Limited financial resources often leave schools with outdated infrastructure and facilities.

The second component, infrastructure, encompasses the necessary elements for technology implementation, including ICT hardware, software, facilities, and network systems [28]. Access to ICT resources is therefore essential for integrating technology into the educational process [29,30]. The effective integration of ICT in teaching depends on the availability and accessibility of computers, up-to-date software, and reliable hardware [31].

The third component of the model refers to people. It pertains to the crucial role individuals play in integrating technology into teaching and learning practices. Several studies indicate that teachers’ attitudes toward ICT significantly affect its usage [32]. According to [33] and [34], these attitudes and beliefs are more critical for ICT integration in classrooms than other factors. That is, pedagogical beliefs and teacher expertise are essential for effectively integrating ICT into teaching practices because they can either facilitate or hinder its use [35–37]. Similarly, [38] emphasize the importance of teachers’ technical competencies in shaping instructional methods. Furthermore, meeting the needs of all stakeholders while enhancing service quality remains crucial [39].

The model’s fourth component is policy. [40] highlighted that the policy aspect of the RIPPLES model addresses the need for institutions to establish policies mandating the use of technology by their members. Policies are seen as strategic frameworks for transformation, motivating change and coordinating efforts [41]. [42] noted that school policies significantly affect ICT use in classrooms by shaping teachers’ perceptions and influencing ICT integration [43]. [44] emphasized that adequate policies and strategic planning are crucial for sustainable ICT integration. Therefore, a comprehensive framework for strategic and operational planning is vital to effectively direct the use of ICT in education [32].

The fifth component of the model, learning, underscores the importance of technology in enabling institutions to achieve their educational objectives [45]. Governmental entities, including educational institutions, must stay abreast of emerging technologies in today’s dynamic and interconnected global landscape. [39] argue that technology can enhance institutional performance, facilitate the attainment of objectives, deepen individuals’ understanding of their environment, and support effective problem-solving.

Evaluation is the sixth element of the model, referring to the need for continuous assessment of technology. Technology integration into teaching and learning should be systematically and regularly evaluated [46]. According to [47], Monitoring and evaluation are critical for achieving effective ICT application in education. They recommended that schools using ICT should periodically assess their operations to ensure the effectiveness of teaching and learning. To achieve sustained technology integration, school leaders must understand, support, and assess ICT use, recognizing that it represents a transformation in teaching and learning approaches rather than merely a focus on technology itself [48]. Administrators should also be able to evaluate and respond to technology-related issues, ideas, and suggestions [49].

Teachers require both administrative and technical support to integrate technology into their instruction effectively. Different levels of administrative support and leadership in technology influence the success of ICT integration in education [50]. Support from school principals encourages teachers to adopt ICT in their instructional practices [51]. Moreover, technical assistance positively affects how educators use and integrate ICT into their teaching and learning processes [52,53]. Successful ICT integration, therefore, depends on the availability of technical support throughout the curriculum; without it, educators may encounter challenges and diminished motivation to use technology [54]. Consequently, it is essential to provide direction, assistance, and services as part of technology implementation efforts [55].

## 2. Problem statement

Cognizant of the importance of technology in education, the government of Ethiopia has been making significant investments in ICT. [56], for instance, note that the government has launched strong and ambitious initiatives to promote ICT in education. However, evidence from various sources indicates that multiple challenges constrain the effectiveness of ICT utility in the secondary schools of the current study area. For example, [57] revealed that obstacles exist in effectively integrating ICT in schools. Similarly, [58,59], and [60] noted that ICT is not sufficiently utilized in Ethiopian schools to enhance educational quality. Consistently, although the authorities are well aware of ICT’s potential to improve schooling outcomes, the Amhara Bureau of Education report [61] states that the majority of its secondary schools experienced limitations in the use of ICT in teaching and learning. These findings underscore the urgent need to identify the critical factors hindering ICT integration, a necessary step toward developing targeted, practical solutions.

Furthermore, data from the Amhara Bureau of Education (ABoE) indicated that student performance in grades 9–12 has declined over the last five years, as reflected in both national exams and school-level assessments, across the majority of secondary schools. While multiple factors contribute to it, the inadequate integration of ICT into teaching and learning is often recognized as a key factor in student performance.

Therefore, the researchers identified the following critical gaps that this study aimed to address: First, rather than being examined comprehensively, elements that could affect teachers’ use of ICT and their activities are typically discussed separately. Second, due to variations in population, economic development, and cultural background, influencing factors can differ between nations. Third, previous studies have overlooked critical variables crucial for achieving learning objectives. This study, therefore, aims to investigate whether and to what extent RIPPLES-related factors influence the effective use of ICT in public secondary schools in the Amhara region of Ethiopia. The following research questions guided the entire study:

1. To what extent does each RIPPLES factor explain the utility of ICT in the context of the current study area?
2. Is there a significant difference between the perceptions of teachers and students regarding the utility of ICT in their instructional practices?

## 3. Materials and methods

### 3.1. Study design

That is because this design helps identify and measure the strength and direction of relationships across the factors specified in the RIPPLES model and the utility of ICT in the current study area, without manipulating them [62]. If the antecedents of ICT utility are strongly related to ICT use, this allows for making reasonable predictions or estimates of the other [63,64]. Additionally, correlational designs often serve as a foundation for further experimental research by revealing potential causal relationships worth exploring [65].

### 3.2. Population and sampling

The study involved public secondary school teachers and students in the state of Amhara. There were 40,610 teachers and 537,809 students, spread across 659 public secondary schools in the state [66]. Using a multistage sampling technique, the study involved four zonal administrations and two regio-polytan cities. Among them, 13 woredas were involved, of which 23 secondary schools were selected using proportionate stratified random sampling. Thus, with a precision of 0.05, the calculated sample sizes were 244 teachers and 246 students. However, because multistage sampling was used, a larger sample size was required to achieve the same level of precision. To adjust for this, the researchers multiplied the sample size by the design effect [67]. Considering a 10% non-response rate, the final sample sizes of 805 teachers and 811 students were selected using simple random sampling.

### 3.3. Data collection instruments

Considering the nature of the research questions, data were collected using a survey questionnaire. This method was chosen because it allows for the efficient collection of substantial primary data from a large number of participants within a limited timeframe and budget [68,69]. [70] questionnaires can also enhance respondents’ honesty due to the anonymity they provide, while remaining cost-effective. To ensure the validity and relevance of the instrument, the researchers adapted items from [71] on the effectiveness of ICT integration in educational settings and from [44], which explored the factors affecting the effective use of ICT.

### 3.4. Instrument validity, reliability, and model fit tests

The researchers implemented several procedures to ensure the validity of the customized data collection instruments, emphasizing face, content, discriminant, and construct validity. To that end, the questionnaire was piloted before its full-scale implementation. Faculty members from Bahir Dar University, specialists in Computer Science, Educational Technology, Psychology, Curriculum and Instruction, and Language and Literature, were invited to review each item and provide detailed feedback based on their expertise. Experts’ evaluation reports on the relevance of each item were measured using [72]’s Content Validity Ratio (CVR) model, and only those items with a CVR of 0.62 or higher were retained. Furthermore, CFA were conducted, and their outputs indicated strong convergent validity (factor loadings exceeding 0.5) and significant discriminant validity, confirming that the constructs were conceptually distinct and free from double loading, as stated by scholars in the field [73,74].

**Fig 1:**
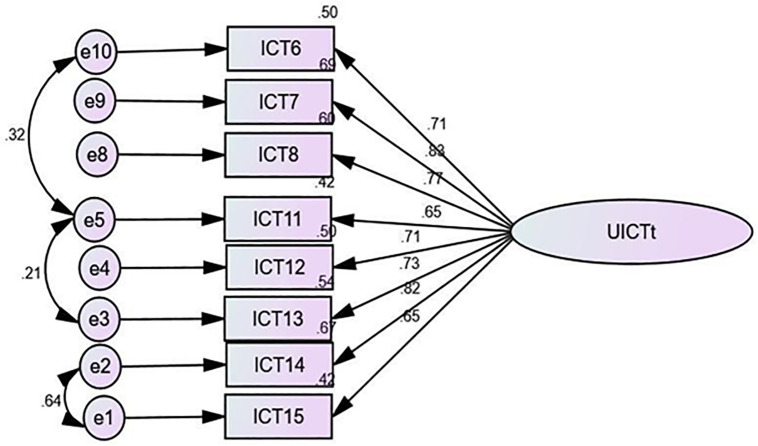
Confirmatory Factor Analysis for Utility of ICT (Teachers’ Perspective).

**Fig 2:**
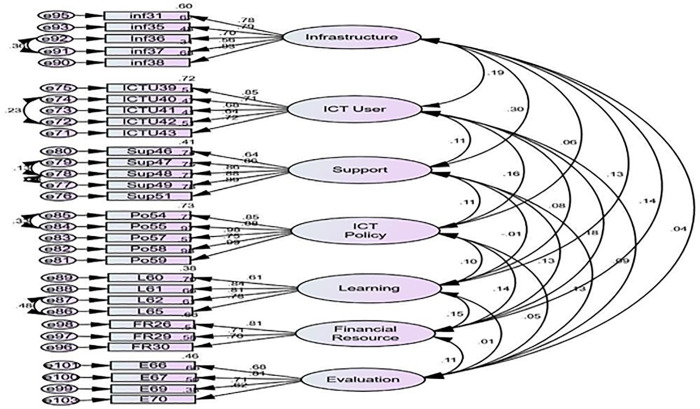
Confirmatory Factor Analysis for Antecedents of Utility of ICT (Teachers’ Perspective).

The CFA results in Fig 1 showed the model structure and standardized parameter estimates. In other words, based on teachers’ perspective, all factor loadings for the utility of ICT ranged from 0.65 to 0.88 in standardized regression weights. Similarly, as shown in Fig 2, based on students’ perspectives, the range is 0.68–0.77. This indicated that the results of factor loadings exceeded the desirable standard of 0.50 [74].

The Model Validity Measures composite reliability, Average Variance Extracted, and the Maximum Reliability of the hypothesized factor model, MaxR(H), for the utility of ICT from the teachers’ perspective were 0.904, 0.543, and 0.912, respectively. And 0.892, 0.508, and 0.894 from the students’ perspective. This result suggests that the hypothesized factor model is reliable, indicating that the factors are well-defined and the indicators are strongly associated with their corresponding latent constructs. According to the indices provided in Table 1, the models for teachers and students demonstrate an excellent fit. All the fit indices (GFI, TLI, CFI, and RMSEA) for both groups exceed the acceptable ranges, indicating that the model fits the data well for teachers and students.

**Table 1:** Fit Measures for The Measurement Model for the Utility of ICT.

	Groups	χ2/df	GFI	TLI	CFI	RMSEA
Model	Teachers	4.332	.975	.974	.984	.067
	Students	2.567	.983	.984	.989	.045
Acceptable range		≤ 5	≥.90	≥.90	≥.90	≤.08

Based on the Teachers’ perspective, the CFA results of convergent and discriminant validity of the antecedents of the utility of the ICT scale in Table 2 revealed that the AVE value of each construct exceeded 0.5; this indicates that each construct (or latent variable) explains more than 50% of the variance in its observed indicators. So, it indicates good convergent validity [75]. Moreover, the values in bold on the diagonal in Table 2 indicate that the square root of the AVE for each scale dimension surpassed the maximum shared value and the inter-variable correlation. Indicating that constructs were independent, devoid of double-loading indicators, and had strong discriminant validity [73]. Table 2 shows that the composite reliability values for the antecedents of ICT utility ranged from 0.81 to 0.91, exceeding the acceptable threshold of 0.7, indicating robust internal consistency of the measurement scales.

**Table 2:** Model Validity Measures of Antecedents of Utility of ICT for Teachers’ Perspective.

	CR	AVE	MSV	MaxR(H)	1	2	3	4	5	6	7
ICTUser	.844	.522	.035	.861	**.723**						
Support	.917	.691	.092	.931	.110**	**.831**					
Policy	.953	.803	.025	.988	.158***	.105**	**.896**				
LIICT	.848	.586	.021	.866	.076	−.015	.102*	**.766**			
Infra	.858	.552	.092	.878	.187***	.304***	.052	.131**	**.743**		
Fresource	.807	.583	.031	.813	.175***	.129**	.143***	.146**	.130**	**.764**	
Evaluation	.799	.500	.017	.815	.095*	.129**	.049	.007	.039	.109*	**.707**

*Note: CR = composite reliability, AVE = average variance extracted, MSV = maximum shared variance, MaxR(H)= maximum reliability of the hypothesized factor model, Fre = financial resource, Infra = infrastructure, ICTU = information and communication technology user, LIICT = *Learning the impact of *information communication technology.*

**Fig 3:**
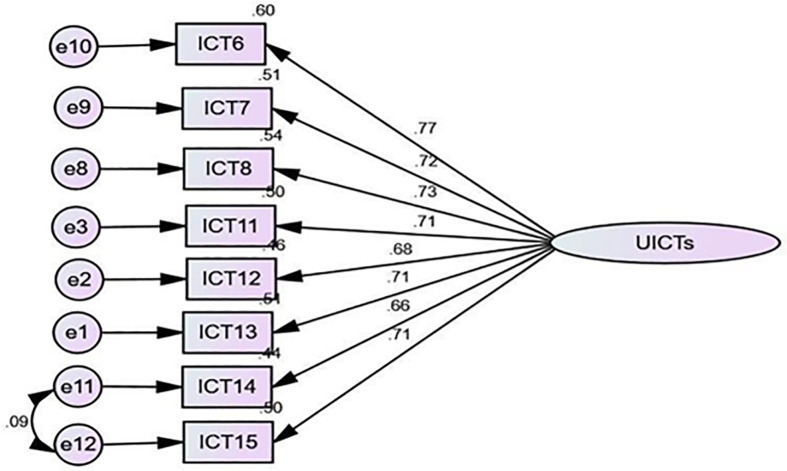
Confirmatory factor analysis for utility ICT (Students’ perspective).

**Fig 4:**
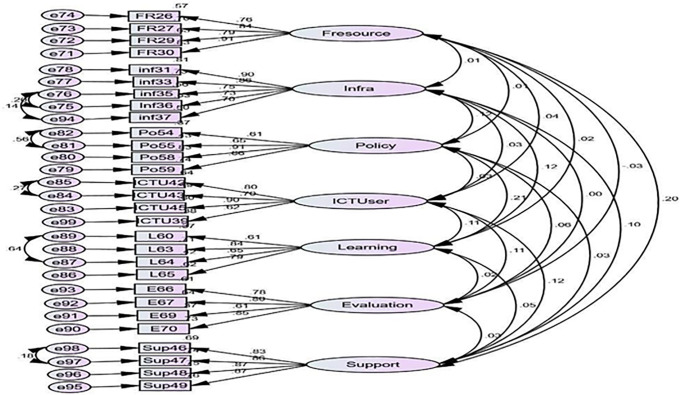
Confirmatory Factor Analysis for Antecedents of Utility of ICT (Students’ Perspective).

Moreover, the CFA results in Figs 3 and 4 showed that the measurement model of antecedents of UICT differentiates between observed and latent variables. The figures show the model structure and standardized parameter estimates. In other words, as shown in Fig 3, the factor loadings of the seven sub-scales for the teachers’ perspective ranged from 0.61 to 0.98 in standardized regression weights, and the results in Fig 4 revealed that they ranged from 0.61 to 0.91 for the students’ perspective. It indicated that the results of factor loadings exceeded the desirable standard of 0.50 [74].

As depicted in Table 3, except for the students’ GFI value of 0.897, which is closer to the target of 0.9, all the other fit indices were within the acceptable range. These results indicate that the proposed research model is a good fit.

**Table 3:** Fit Measures for The Measurement Model of Antecedents of Utility ICT.

	Participants	χ^2^/df	GFI	TLI	CFI	RMSEA
Model	Teachers	2.979	.905	.938	.946	.052
	Students	3.554	.897	.920	.931	.058
Acceptable range		≤ 5	≥.90	≥.90	≥.90	≤.08

From the student’s perspective, the CFA results for the convergent and discriminant validity of the antecedents of the ICT scale’s utility, presented in Table 4, indicated that each construct’s AVE exceeded 0.5, indicating good convergent validity. This means that the observed variables (indicators) are highly correlated with the latent construct, and the construct explains a significant portion of the indicators’ variance, confirming the scale’s convergent validity [73,76].

**Table 4:** Model Validity Measures of Antecedents of the Utility of ICT for Students’ Perspective.

	CR	AVE	MSV	MaxR(H)	1	2	3	4	5	6	7
Fresource	0.895	0.682	0.039	0.910	**0.826**						
Infra	0.892	0.624	0.015	0.911	0.010	**0.790**					
Policy	0.850	0.592	0.044	0.900	0.009	0.121**	**0.769**				
ICTUser	0.845	0.581	0.014	0.882	0.041	0.029	0.032	**0.762**			
LIICT	0.816	0.530	0.044	0.844	0.021	0.121**	0.210***	0.114**	**0.728**		
Evaluation	0.848	0.585	0.012	0.868	−0.028	−0.004	0.060	0.111**	0.024	**0.765**	
Support	0.918	0.737	0.039	0.919	0.197***	0.096*	0.030	0.120**	0.055	0.031	**0.858**

The bolded diagonal values in Table 4 showed that the square root of the AVE for each scale dimension exceeded both the maximum shared variance and the inter-variable correlation. The requirement is that the square root of the AVE or the diagonal bold values should be higher than the other values in their respective rows and columns, which is met, as shown in the table. Suggesting that constructs were unrelated or contained no double-loading indicators and showed good discriminant validity [73]. As shown in Table 4, the composite reliability values of antecedents of the utility of ICT ranged from 0.81 to 0.91, exceeding the acceptable level (0.7) and indicating good internal consistency of the measurement scales.

### 3.5. Method of data analysis

The researchers conducted preliminary analyses and found no violations of the normality, linearity, and homoscedasticity assumptions. This study employed the mean, independent samples t-test, and multiple linear regression to analyze the data. Mean scores and the independent-samples t-test were used to assess teachers’ and students’ perceptions of the utility of ICT in the teaching–learning process and whether the two groups differ significantly in their perceptions, whereas regression analysis was used to examine the extent to which each factor explained ICT utility. Data analysis was conducted using SPSS Version 27 and AMOS Version 23.

### 3.6. Ethical consideration

This survey data was collected for research from February 20 to March 20, 2025. The Institutional Review Committee for Research and Community Services, located in the Vice President’s Office of Bahir Dar University, reviewed and approved the study, assigned number 022 and Protocol number 019. During the data collection, the researchers were dedicated to show due respect to both the research site and the individuals involved. Initially, we secured verbal approval and consent from the school administrators to collect data from teachers. Regarding students’ informed consent, the Institutional Review Board (IRB) waived it since the research focus was not age-sensitive. The entire data collection process, including verbal consent, was approved by the IRB and supported by a letter dated September 26, 2025, BDUCOEIRRC/35/2025.

Additionally, we were open, genuine, and straightforward in communicating information to participants, ensuring they had a comprehensive understanding of the study’s goals and our role within it. The choice to use verbal consent stemmed from practical reasons: participants frequently decline to participate if asked for written consent. Even those who agree to sign often feel hesitant to provide accurate information when a written consent is required.

## 4. Results

### 4.1. Return rate

The study surveyed 805 teachers and 811 students using questionnaires. Of these, 766 teacher questionnaires and 789 student questionnaires were returned. During data cleaning, 27 teacher and 31 student questionnaires were discarded due to incompleteness. Ultimately, 739 questionnaires from teachers (91.8%) and 758 from students (93.5%) were used for data analysis. This response rate was considered adequate to proceed with the analysis phase, as scholars in the field, such as [77], suggest that a return rate of ≥70% is acceptable.

### 4.2. Roles of RIPPLES on UICT

Table 5 demonstrates the correlation statistics among the RIPPLES and the utility of ICT in schooling practices. The figures in the table depict a positive relationship between the antecedents specified in the model and the utilization of ICT in instructional practices. Although all the factors identified as RIPPLES show a strong positive correlation with ICT utility in instruction, support is found to be the strongest predictor in the context of the current study area (r = .707).

**Table 5:** Correlations Statistics in Teachers’ Views (N = 739).

Variables	1	2	3	4	5	6	7	8
1. UICT	1							
2. Fresource	.489^**^	1						
3. Infrastructure	.506^**^	.304^**^	1					
4. ICT User	.465^**^	.322^**^	.285^**^	1				
5. Support	.707^**^	.385^**^	.390^**^	.317^**^	1			
6. Policy	.344^**^	.266^**^	.155^**^	.228^**^	.253^**^	1		
7. LIICT	.345^**^	.284^**^	.254^**^	.166^**^	.206^**^	.154^**^	1	
8. Evaluation	.329^**^	.251^**^	.160^**^	.178^**^	.239^**^	.142^**^	.126^**^	1

**Values are significant at the 0.01 level (2-tailed).

### 4.3. The contribution of antecedents to the utility of ICT

Two separate multiple regression analyses were conducted to disaggregate the effects of antecedents on utility concerning teacher and student groups. As shown in Table 6, significant models emerged for both teacher and student groups (Teachers: F_(7,731)_=204.42, p < .001; Students: F_(7,750)_=120.57, p < .001).

**Table 6:** RIPPLES model fit ANOVA Table.

Model	Group		Sum of Squares	Df	Mean Square	F	Sig.
1	Teachers	Regression	122.986	7	17.569	204.424	.000^b^
		Residual	62.826	731	.086		
		Total	185.812	738			
2	Students	Regression	98.922	7	14.132	120.577	.000^b^
		Residual	87.900	750	.117		
		Total	186.822	757			

^a^Dependent Variable: UICT.

^b^Predictors: (Constant), Evaluation, LIICT, Policy, ICT User, Infra, Financial Re, Support.

Both models presented in Table 7 indicate that the predictors significantly account for the variance in the dependent variables for both students and teachers. An adjusted R2 of 0.659 and 0.525 suggests that efforts to enhance these factors may considerably boost ICT usage, which, in turn, could improve the quality of education. However, the teacher model shows greater variance, suggesting that the predictors are more effective at explaining teacher outcomes than student outcomes. The results reveal that the relationship between antecedent factors and ICT usage differs between student and teacher populations.

**Table 7:** Effects of predictors of the RIPPLES model on UICT.

Model	Group	Variables	Unstandardized Coefficients		Standardized Coefficients	T	Sig.	Adjusted R Square
			B	Std. Error	Beta			
1	Teachers	(Constant)	−.187	.092		2.027	.043	.659
		Financial Re	.098	.021	.118	4.681	.000	
		Infra	.170	.023	.180	7.393	.000	
		ICT User	.139	.020	.170	7.138	.000	
		Support	.409	.022	.465	18.395	.000	
		Policy	.081	.019	.096	4.215	.000	
		LIICT	.091	.018	.115	4.995	.000	
		Evaluation	.085	.019	.101	4.454	.000	
2	Students	(Constant) Financial Re	−.273	.117		2.342	.019	.525
			.115	.021	.142	5.439	.000	
		Infra	.080	.022	.096	3.700	.000	
		ICT User	.351	.026	.368	13.274	.000	
		Support	.224	.023	.266	9.607	.000	
		Policy	.069	.026	.071	2.656	.008	
		LIICT	.122	.024	.140	5.117	.000	
		Evaluation	.122	.020	.160	6.222	.000	

^a^Dependent Variable: UICT.

The regression coefficients for the teachers’ group indicate how each predictor impacts UICT: F_(7, 731)_ = 204.424, p < 0.001. The values of the standardized coefficients of determination shown in Table 8 are (.118,.180,.170,.465,.096,.115, and.101) at p < 0.05 for financial resource, Infrastructure, ICT user, support, policy, LIICT, and evaluation, respectively. Similarly, for the student group, the regression coefficients provide insight into the antecedents of UICT: F_(7, 750)_ = 120.577, p < 0.001. The values of the standardized coefficients of determination are (.142,.096,.368,.266,.071,.140, and.160) at p < 0.05 for financial resources, Infrastructure, ICT user, support, policy, LIICT, and evaluation, respectively.

**Table 8:** Independent Sample t-test between teachers’ and students’ perceptions of the utility of ICT in instructional practices.

Levene’s Test for Equality of Variances	F	Sig.	t-test for Equality of Means				Cohen’s d
			T	df	Sig. (2-tailed)	Mean Difference	
Equal variances assumed	.017	.897	2.771	1495	.006	.07162	0.143
Equal variances not assumed.			2.771	1492.349	.006	.07162	

### 4.4. Perceptions of participants on the UICT in instructional practices

An independent-samples t-test was conducted to examine teachers’ and students’ perceptions of the utility of ICT in instructional practices. The mean score for teachers was M = 2.894, whereas that of students was M = 2.822. Both mean values are below average, indicating a general lack of certainty regarding the utility of ICT for teaching and learning among both groups. The t-test results (see Table 8) revealed a weak but statistically significant difference in perceptions between teachers and students (t = 2.771, d = 0.143).

## 5. Discussions

### 5.1. Relationship between antecedents and utility of ICT

#### 5.1.1. Support and Infrastructure.

The results showed a positive correlation between support (administrative, technical, and pedagogical) and UICT in teaching practices (r = 0.707). Similarly, infrastructure, including hardware availability, software, and network installation, showed a positive relationship with UICT (r = .506). This result aligns with the findings of several researchers [52,53,78–81], who also reported a positive correlation between different forms of support and the effective integration of ICT in teaching practices. Similarly, the work of [82,83], and [84] highlighted that ICT infrastructure is a key factor influencing technology, thereby significantly enhancing instructors’ pedagogical use of ICT in education.

#### 5.1.2. User characteristics.

A positive correlation was observed between ICT users (r = 0.465) and the availability of financial resources (r = 0.489 This result was consistent with the finding of [27], who revealed that integrating technology into educational institutions in most developing countries is challenging due to the need for substantial public funding. Likewise, [84] and [37] identified a positive correlation between teachers’ ICT proficiency and the use of ICT for educational purposes. This indicates that teachers interested in ICT will also be eager to incorporate it into the classroom.

#### 5.1.3. ICT Policy and Learning the Impact of ICT.

ICT policy (r = 0.344) and Learning the Impact of ICT or LIICT (r = 0.345) showed a positive relationship with UICT. This result was consistent with [42], who found that policy shapes teachers’ perceptions by influencing the integration, organization, and implementation of ICT in education. Similarly, the study by [42] examined the effect of school policies on ICT integration in classrooms. It found that explicit policies lead to the regular use of ICT. Teachers believe that ICT integration benefits them when they are well-informed about technology use. The findings of this study were consistent with those of [45], who emphasized that successful technology adoption requires administrators and teachers to view technology as a tool that enhances teaching and learning. Furthermore, [85] found that teachers must have a profound understanding of the educational role of ICT to integrate it into their instructional approaches successfully.

#### 5.1.4. Evaluation.

Finally, a positive correlation (r = 0.329) was observed between evaluations and UICT. The findings of this study are consistent with the fact that effective technology leadership will implement assessment protocols to gain insights from continuous experience [86]. Similarly, [47] confirmed that Monitoring and evaluation are critical for achieving ICT implementation in education. They recommended that educational institutions using ICT for education should periodically evaluate their operations to ensure effective teaching and learning activities.

Therefore, it is possible to conclude that a positive relationship exists between the listed independent variables in the RIPPLES model and UICT in teaching and learning practices.

### 5.2. Impact of antecedents on UICT

The first research question we sought to answer in this study was: To what extent does each factor explain the utility of ICT in the current study area? The results of our multiple linear regression model showed that each predictor in the RIPPLES model affects UICT. Various factors appear to influence the integration of ICT into teaching practices, include difficulties related to insufficient Infrastructure [82,87], technical and administrative support [88], teachers’ and students’ attitudes, knowledge, and interests [31,38,89], policies and plans [32,78,90], and evaluation [48,91]. Overall, these findings are consistent with our results.

Support (technical and administrative) has the most significant standardized coefficient (0.465). For each one-unit increase in Support, UICT increases by 0.409 units. This is the most influential teacher predictor, with a strong positive relationship with UICT and a statistically significant p-value < 0.001. This result is consistent with that of different researchers. Administrative support encourages ICT integration through role models, such as principals, who need to attain ICT proficiency to provide effective technological leadership across administrative, instructional, and learning tasks [78,79]. The authors also emphasized the importance of transformational leadership, which can help enhance the integration of ICT into teaching and learning processes. Consequently, the availability of administrative support is crucial for encouraging teachers to use computers in their teaching [53].

In addition, [91] reported that technical assistance typically encompasses help desks and online support services that assist users in accessing, operating, and resolving issues related to hardware, software, and network resources [52]. This suggests that many teachers are primarily concerned with continuous support despite the undeniable importance of ICT training. [55] also noted that providing guidance, support, and services is essential for the effective use of technology. This implies that classroom teachers cannot effectively cultivate the confidence, skills, and expertise required to incorporate ICT into their instructional approaches without appropriate technical assistance.

ICT users (in terms of their interests, attitudes, skills, and knowledge) have the strongest positive relationship with UICT in the students’ model. For each unit increase in ICT Users (their attitude, skill, interest, knowledge, and competence), UICT increases by 0.351 units, making this the most influential predictor for students and statistically significant (p-value < 0.001). Additionally, this finding aligns with previous evidence demonstrating the beneficial impact of attitudes on the integration of ICT [92–94]. ICT users’ attitudes toward technology and perceptions of technology integration in teaching and learning practices strongly influence teachers’ and students’ use of instructional strategies and technology integration [92,95].

Furthermore, ICT competency significantly impacts the utilization of ICT [96]. It is likely that students with a more favorable attitude toward ICT exhibit greater enthusiasm for acquiring new skills and demonstrate greater perseverance in applying new knowledge, even in the face of challenges. This suggests that when ICT users believed they were adequately proficient, they reported increased ICT use. The frustration resulting from the inability to utilize ICT likely hinders users from employing technology widely in their teaching and learning practices.

The study also found that ICT policy had the weakest positive effect on UICT among teachers and students in the public secondary schools of the state of Amhara. This finding is consistent with [97], who noted that students, teachers, and even institutional leaders often lack access to ICT policy, making it difficult to compare their institutions’ ICT practices with the government’s educational ICT plans and policies. Similarly, [98] found that many schools lack ICT policies, making it harder for students to learn to use these tools properly and to meet the school’s goals and students’ needs. Furthermore, [44] revealed that effective policies and strategic planning are necessary for sustainable ICT integration. This suggests that having a well-defined policy framework can foster a school culture that promotes the use of ICT.

### 5.3. Perception of teacher and student

The second research question we sought to answer in this study was: Is there a significant difference between teachers’ and students’ perceptions regarding the utility of ICT in their instructional practices? Our survey findings indicated that teachers’ perceptions of the utility of ICT in instructional practices are slightly higher than those of students. In line with this study’s findings, [99] argue that students and teachers hold divergent viewpoints regarding the implementation of ICT in pedagogy and learning. The intensity of ICT use varies for various reasons, including education, entertainment, and socio-economic activities. [100] found a difference in how students and teachers use ICT devices for social networking. According to these authors, teachers utilize ICT devices more extensively for personal and academic purposes than students do.

Similarly, a study by [101] reveals that teachers and students hold differing views on the impact of ICT on learning and teaching. The study’s findings showed that students thought ICT had a moderate effect on their learning. According to school teachers, the results were unique; they believed ICT had a significantly greater impact on teaching [101]. [102] argue that students tend to use ICT primarily for recreational purposes, and that their academic use is often limited or inefficient. This suggests that, despite their intense engagement with technology, students’ primary focus is on socializing and entertainment rather than on learning. [103] also suggests that while students might use ICT more frequently for personal reasons, teachers use ICT with more intent and skill for educational purposes.

## 6. Conclusion, implications, and recommendations

### 6.1. Conclusion

The study revealed that all antecedents in the RIPPLES model positively and significantly influence the utility of ICT in instructional practices. Among these, support emerged as the strongest predictor for teachers, while ICT user characteristics were the most influential for students. Infrastructure, financial resources, policy, learning impact of ICT, and evaluation also contributed meaningfully to ICT utilization. Despite these positive associations, both teachers and students exhibited below-average perceptions of ICT’s utility, indicating uncertainty and limited confidence in its pedagogical value. Overall, it can be concluded that enhancing technical and administrative support, user competence, and infrastructural capacity, along with effective policy implementation and evaluation, can substantially improve ICT integration and the quality of teaching and learning.

### 6.2. Implications

The findings of this study provide important insights into the factors that influence the use of ICT in public secondary school instruction. These insights have significant policy implications and practical implications outlined below. From a policy viewpoint, the research highlights the need for evidence-based strategies to integrate ICT. Decision-makers should think about establishing ICT integration in secondary education. This should be supported by clear rules and guidelines that cover various aspects. Understanding what drives ICT use will help school leaders and policymakers make better investment decisions, rather than assuming that just providing devices will lead to successful integration. From a practical perspective, the results stress the importance of training teachers to use ICT as a valuable tool for improving education. Teachers need to incorporate ICT tools into their teaching methods to enhance student learning outcomes, motivation, engagement, and skill development. This integration should align with curriculum goals and classroom realities to ensure it is relevant and effective.

### 6.3. Recommendations

The results provided a deeper understanding of the factors contributing to the underutilization of ICT in education; thus, it is vital to formulate recommendations based on these insights.

- School authorities, right from the Bureau of Education down to the principal, shall prioritize and improve technical and administrative support so that teachers can use computers effectively in day-to-day classroom sessions.
- Woreda and zonal education offices shall provide continuous professional development programs for teachers to help them effectively utilize ICT in their instructional exercises.
- The education bureau needs to establish a suitable ICT infrastructure that aligns with the study area’s context, so schools can harness local knowledge and advance global solutions within their own schools.
- Schools shall develop an ICT policy and strategy that guides the successful implementation of ICT in their day-to-day instructional practices and engages more teachers and students in its use.

## Supporting information

S1 File Teachers’ data. (XLSX)

S2 File Students’ data. (XLSX)
